# Isolated congenital inter-costal pulmonary hernia: a case report

**DOI:** 10.1186/s13256-019-2142-4

**Published:** 2019-07-18

**Authors:** Toussain Wendlamita Tapsoba, Christine Grapin-Dagorno, Arnaud Bonnard, Alaa El-Ghoneimi

**Affiliations:** 1 University Pediatric Hospital Charles de Gaulle, Ouagadougou, Burkina Faso; 2 Robert DEBRE Hospital, Paris, France

**Keywords:** Hernia, Lung, Intercostal, Congenital, Thoracoscopy

## Abstract

**Background:**

Intercostal lung herniation is a rare condition that may be congenital (20%) or acquired (80%). The isolated congenital form is exceptional, with one case reported in the literature.

**Case presentation:**

We report a case of a 10-year-old French boy of Algeria origin, born with intermittent swelling of his right hemithorax. The swelling and pain gradually increased with age. A clinical examination revealed a localized swelling of his right hemithorax at the level of the midclavicular line and the fifth intercostal space. The swelling increased in size during respiratory movements and enlarged with Valsalva maneuvers. The intercostal lung hernia was treated by thoracoscopy.

**Conclusions:**

This is the second case of isolated congenital intercostal pulmonary hernia reported in the French and English literature. It is the first to be treated by thoracoscopy. Based on this case we performed a review of the diagnosis and therapeutic aspect of pulmonary hernias.

**Electronic supplementary material:**

The online version of this article (https://doi.org/10.1186/s13256-019-2142-4) contains supplementary material, which is available to authorized users.

## Introduction

A lung hernia is rare, particularly among children [1, 2]. In 80% of cases, lung hernia is acquired and in 20% of cases, it is congenital [3, 4]. The congenital forms are typically linked to polymalformation of the ribcage [1, 5]; they are exceptionally isolated. In 2004, Chattopadhyay and colleagues described the first case [1].

A consensus has not been reached among physicians regarding the management of lung hernias [6, 7]. This case report will focus on the case referenced as the first treated by thoracoscopy. We will review the diagnosis and therapeutic aspects of this rare pathology.

This clinical case is exceptional because of its isolated congenital nature. All these reasons justified our sharing of our experience because for this rare pathology there is no codified treatment.

## Case presentation

This case report describes the case of a 10-year-old boy of Algeria origin living in Ile-de-France. Due to extremely painful thoracic swelling, the boy’s parents brought him in September 2012 to our hospital. This small nodular swelling was apparent from birth and progressively increased in volume. The chest pain appeared when the boy was 10-years old and his parents consulted for a treatment request at the pediatric hospital Robert Debré. The boy failed to receive treatment prior to this. He had never experienced thoracic trauma and he did not possess any known prior medical pathology. He had no risk of exposure to toxins in his environment. Moreover, no similar case was noted in his family and there is no hereditary disease and no consanguinity between parents.

**Fig. 1:**
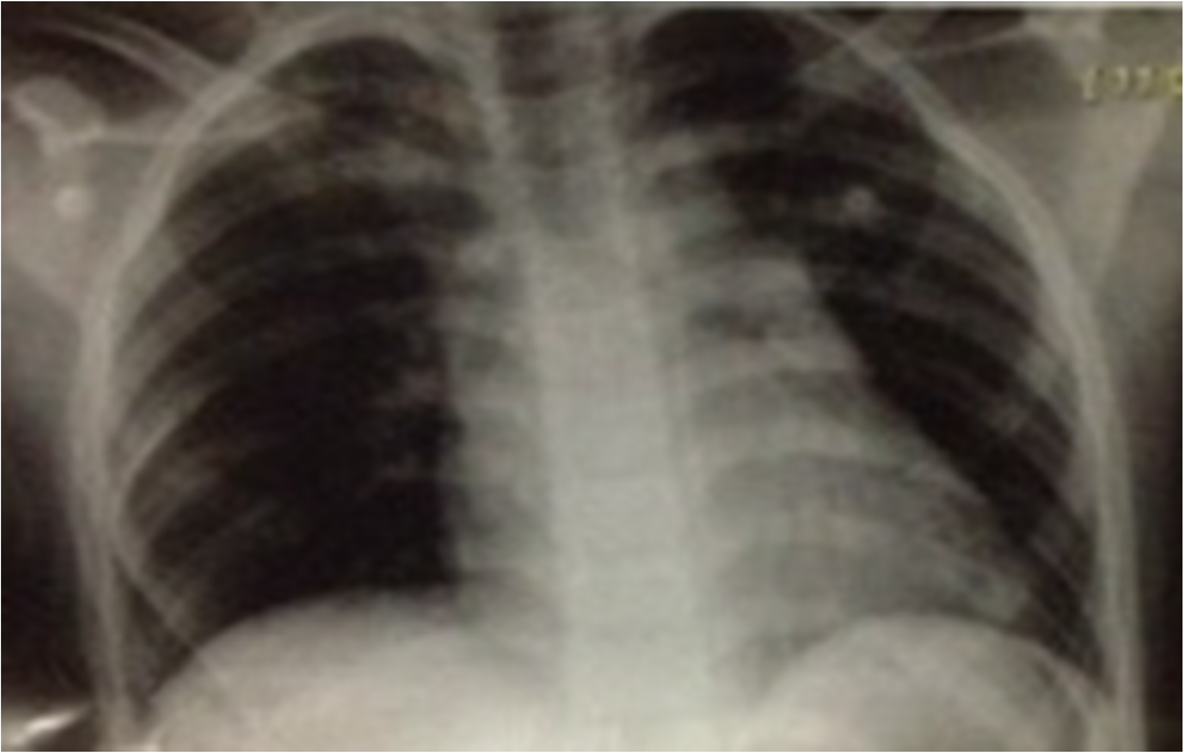
Standard X-ray of the chest (normal)

**Fig. 2:**
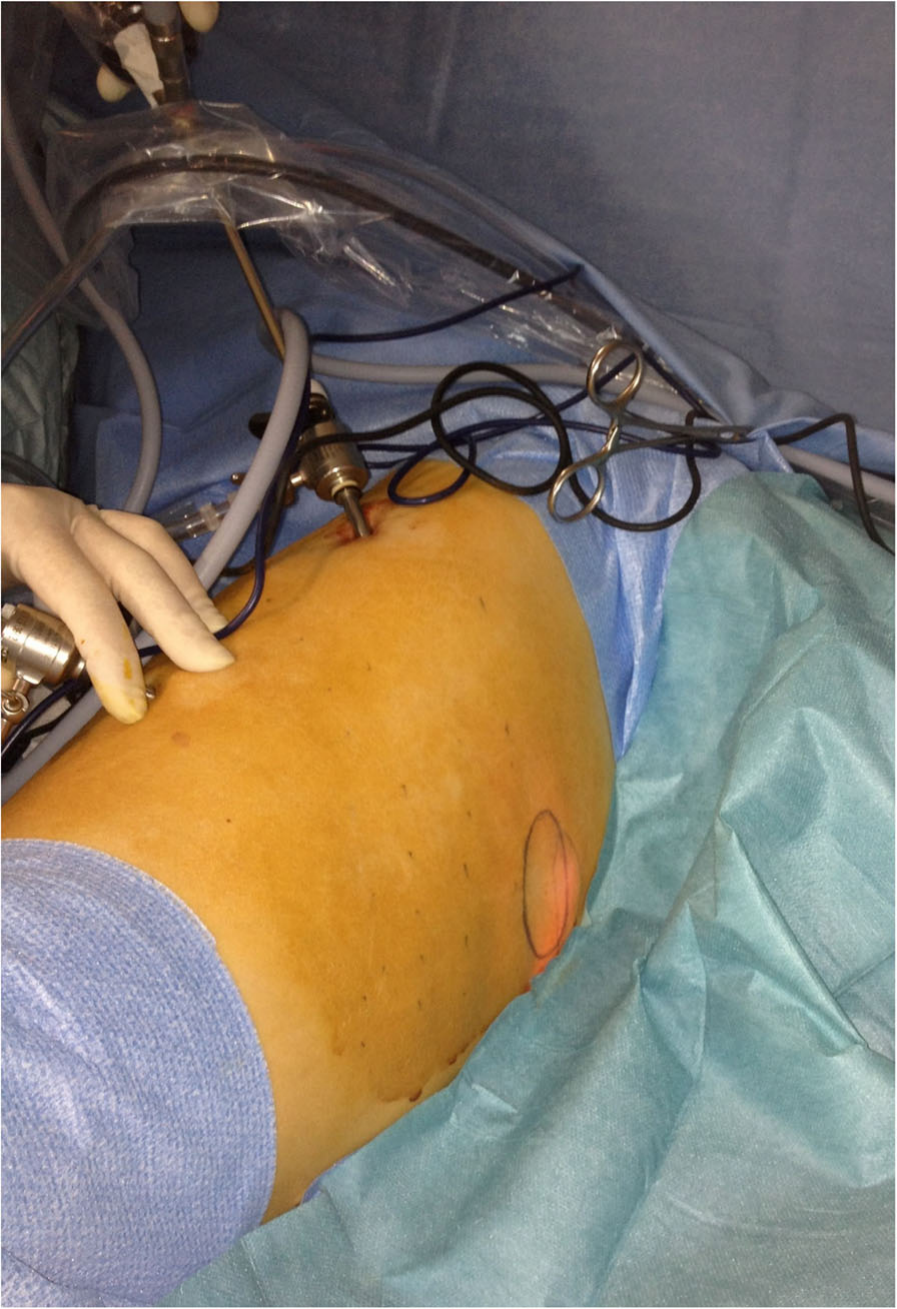
Patient position for right chest thoracoscopy

An initial clinical examination showed a well-developed child with no other physical abnormalities. His weight was 41.7 kg, blood pressure 85/140 mmHg, pulse rate 60 pulses/minute, respiratory rate 15 cycles/minute, and temperature 37 °C. His Glasgow Coma Score was 15/15. His cognitive functions were preserved. Sensitivity, motor skills, and osteotendinous reflexes were preserved in his limbs. There was no motor coordination disorder. There was no sphincter deficit. However, a clinical examination revealed a swelling of the right hemithorax (5 cm × 2.5 cm) on the midclavicular line and the fifth intercostal space. His respiratory movements caused the swelling to vary and enlarge with Valsalva maneuvers. Pulmonary and cardiovascular auscultation was normal. We diagnosed a congenital intercostal lung hernia based on the clinical information. A standard X-ray of his chest showed no anomaly for his lungs and thoracic wall (Fig. 1). Laboratory findings showed hemogram, blood serum ionogram, serum creatinine, and liver function within normal range. Given the symptoms, we determined a surgical treatment was most appropriate. Two thoracic and vascular specialty pediatric surgeons performed this with a right-sided chest thoracoscopy. Under general anesthesia, our patient was placed in a left lateral position (Fig. 2) and a 5 mm camera port was inserted in the sixth intercostal space on the posterior axillary line. Two working ports were also inserted: one in the sixth intercostal space behind the posterior axillary line and the second in the ninth intercostal space on the posterior axillary line.

**Fig. 3:**
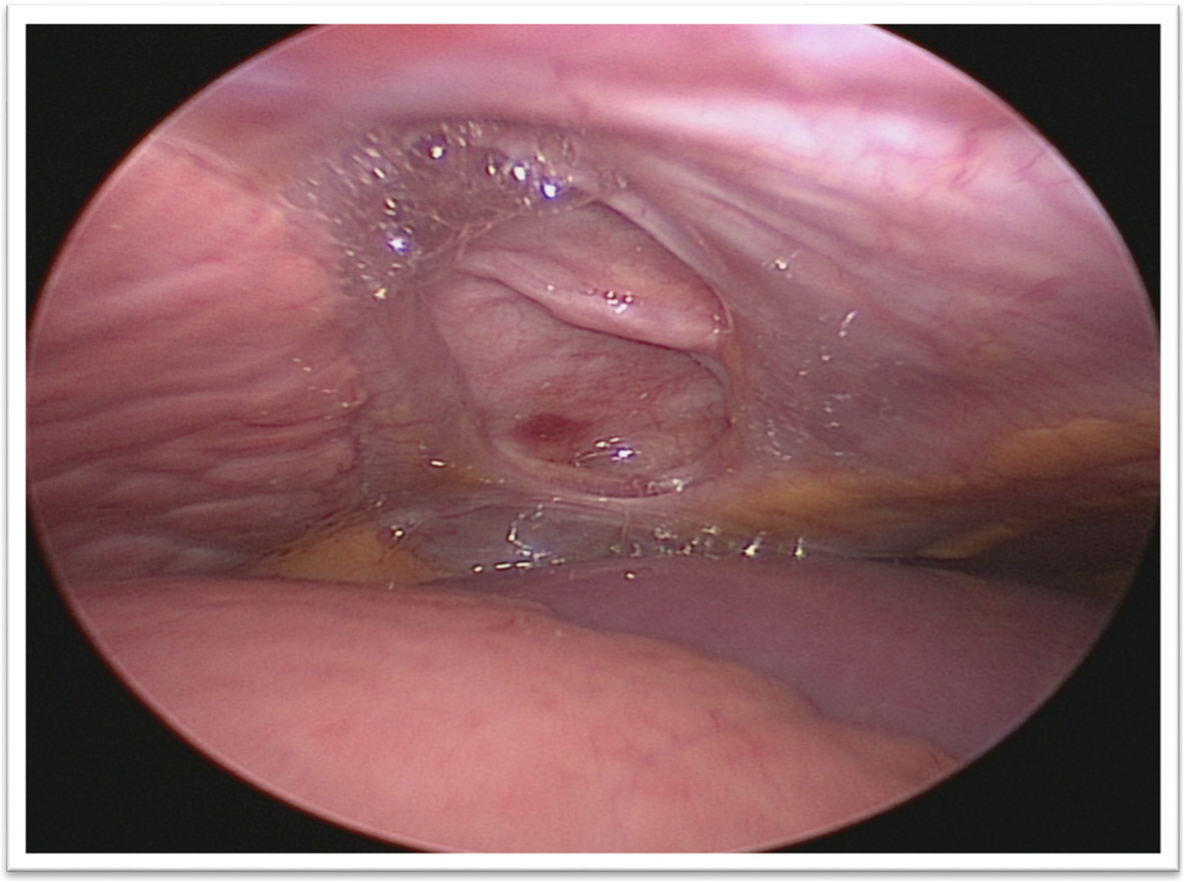
Intercostal muscular and fascial defect in thoracoscopy

**Fig. 4:**
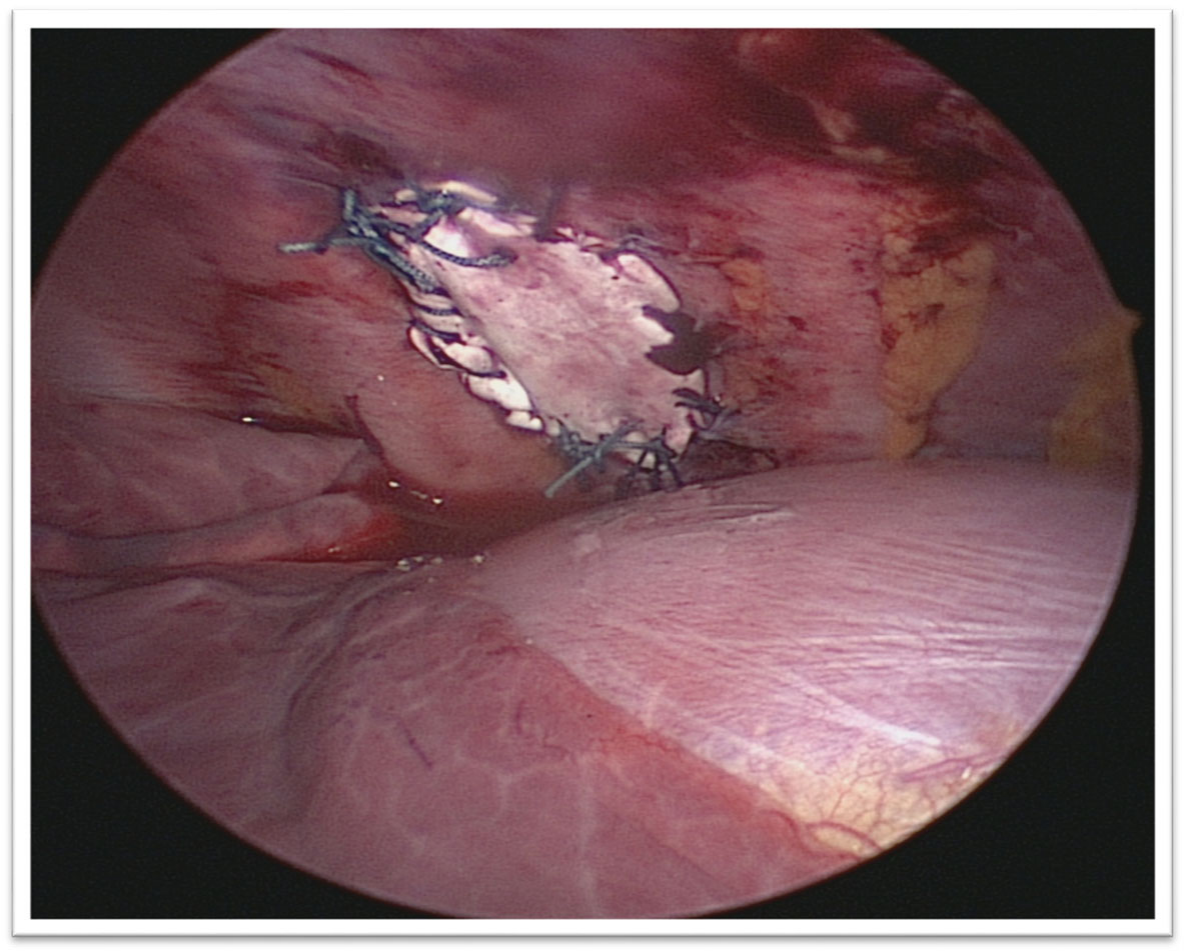
Image of hernia treatment by interposing a polytetrafluoroethylene plate

The camera revealed an intercostal defect consisting of a muscular and aponeurotic aplasia of 4 cm × 2 cm, covered by the parietal pleura (Fig. 3). A polytetrafluoroethylene (PTFE) mesh was inserted to close the defect without incising the hernial sac (Fig. 4). Two semi-continuous sutures were performed with Mersuture 2/0 (Additional files 1, 2, 3, 4, 5, 6, 7 and 8). No complications occurred and a thoracic drain was placed for 48 hours. The repeated clinical and radiographic controls were normal after 1, 3, 6, and 12 months.

## Comments and discussion

This reported case is exceptional. The isolated congenital intercostal lung hernia (ICILH) was asymptomatic at first. It was at the age of 10, when chest pain appeared, that the parents consulted for a treatment request. Except for the swelling of the right hemithorax, the clinical examination was normal.

We chose thoracoscopic surgical treatment. This case is unique and the surgical indication is the first case reported in the literature. The sharing of our experience could allow clinicians to discover or better know this pathology.

In 1946, Maurer and Blades defined a pulmonary hernia as a protrusion of the pulmonary tissue enveloped by the pleura through a defect of the thoracic wall [8, 9]. This is a rare pathology, particularly for children: a total of 300 cases have been noted between 1499 (date of the first description by Roland) and 2004 [3, 9]. However, the etiologies of these hernias are diverse, even for children. Morel–Lavallée described their classification in 1845 [1, 8, 9]. On the one hand, it distinguishes congenital pulmonary hernia (20%) from acquired (80%), on the other hand, it distinguishes them according to topography: cervical (17–35%), intercostal (65–83%), or diaphragmatic [3, 9]. Acquired hernias are traumatic, but some spontaneous cases have been reported among patients with some predisposing factors such as a chronic cough, chronic and obstructive bronchopulmonary diseases, neoplasia, chronic use of steroids, and Ehlers–Danlos syndrome [3, 6, 10, 11]. For the congenital forms, an underlying pathology of the ribcage is frequently associated [5]. The cases of cervical ICILH that have been reported are due to a defect of the fascia of Sibson [1]. The only case of thoracic ICILH has been reported by Chattopadhyay *et al*. about an 7-year-old child in 2004 [1].

Our case report is the second case of ICILH in the literature. Information from a clinical examination was sufficient to make the diagnosis of intercostal lung hernia.

ICILH can be silent and undiscovered during a clinical examination or it can show symptoms if the size is big [6]. When small, the physician can be oriented by the parents who will mention a nodular mass occurring during coughing. In this context, the Valsalva maneuver, as we experienced in this case, can be help in highlighting the tumefaction [12].

As some authors reported, a standard X-ray of the thorax is useful to look for associated lesions [4, 8]. Our patient did not present any lung or thoracic wall lesions. If the diagnosis is unclear, a computed tomography (CT) scan of the chest can be obtained [4, 8]. However, CT is mandatory in the context of trauma to systematically look for associated injuries. A chest CT (inspiratory-expiratory) with a pediatric protocol would be of help, while reconstruction of images (three-dimensional) helps the surgeon to prepare for the operation.

Regarding the treatment, for small hernias, the indications are not codified; there are many controversies. One group of authors advocated abstention or a simple strapping while waiting for spontaneous resolution [1, 13–15]. However, there is a risk for the lump to become bigger with time as seen with our patient [2]. For the other group of authors, abstention is reserved for only supra-pulmonary hernias, all intercostal hernias should be treated surgically [7].

We think it is not an emergency and when a small-sized hernia is discovered early, it should just be monitored. Surgery will be indicated in the case of symptoms such as pain, a persistent cough, an episode of strangulation, or hemoptysis [1, 2]. A continuous growth of the hernia size is also an argument for surgical treatment with the aim to prevent possible trauma or ischemia of the herniated lung [1, 6].

The intervention can consist of a thoracotomy [1, 13]. The first ICILH was treated this way [1]. The real inconvenience in this approach is the scar, which is a problem for pre-adolescents. However, thoracoscopy helps address the aesthetic concern [5]. It also decreases the intensity of the postoperative pain and minimizes the risk of deforming sequelae of the thorax.

Treatment of an intercostal hernia by thoracoscopy was described by Van Den Bossche *et al*. in 1999 [16]. This method was first used in children to treat a post-traumatic intercostal hernia [2].

We report here the first case of treatment by thoracoscopy of an ICILH. It allowed an optimal exploration of the endothoracic wall and the pulmonary parenchyma to look for associated anomalies. The defect of the wall was easily reachable for repair as reported by other authors [6].

The closing of the defect in ICILH can be made through a simple suture or, in cases of large collars, by a transplant of fascia lata or prosthesis as in our patient [13].

Related to the evolution, the results are satisfactory in the different series or reported cases. Recurrences are rare, but a long-term follow-up seems to be necessary.

We agree with other authors that in large pulmonary hernias with or without symptoms, the complications of not treating are greater than complications from treatment [6].

## Conclusion

Congenital Intercostal lung hernia is a rare pathology and the isolated congenital form is exceptionally rare. We report the second case of thoracic ICILH in the literature. The diagnosis was made clinically.

Clinical monitoring is recommended in cases of small asymptomatic hernia, but if the hernia becomes larger with or without symptoms, surgical treatment is indicated. We performed the first thoracoscopy for ICILH. It allowed a simple and efficient surgical treatment, without the inconveniences of thoracotomy.

## Additional files

Additional file 1: **Video 1.** Begining of polytetrafluoro-ethylene mech insertion for closing the defect of intercostal hernia in thoracoscopy. (MP4 2866 kb)

Additional file 2: **Video 2.** Sequence 2 of prothesis fixation on intercostal hernia. (MP4 34887 kb)

Additional file 3: **Video 3.** Sequence 3, showing the following fixation of the prosthetic plate on the intercostal hernia. (MP4 48585 kb)

Additional file 4: **Video 4.** Sequence 4 of prothesis fixation on intercostal hernia. (MP4 24653 kb)

Additional file 5: **Video 5.** Sequence 5, end of the first semicontinuous suture of prothesis on intercostal hernia. (MP4 113395 kb)

Additional file 6: **Video 6.** Sequence 6, performing of the second semicontinuous suture of prothesis on intercostal hernia. (MP4 136579 kb)

Additional file 7: **Video 7.** End of the intercastale hernia repair. (MP4 87975 kb)

Additional file 8: **Video 8.** Final thoracoscopic appearance of the intercostal hernia repair by prosthesis. (MP4 1642 kb)

## Data Availability

Not applicable.
